# Desirable cytolytic immune effector cell recruitment by interleukin-15 dendritic cells

**DOI:** 10.18632/oncotarget.14622

**Published:** 2017-01-13

**Authors:** Heleen H. Van Acker, Ottavio Beretta, Sébastien Anguille, Lien De Caluwé, Angela Papagna, Johan M. Van den Bergh, Yannick Willemen, Herman Goossens, Zwi N. Berneman, Viggo F. Van Tendeloo, Evelien L. Smits, Maria Foti, Eva Lion

**Affiliations:** ^1^ Laboratory of Experimental Hematology, Tumor Immunology Group (TIGR), Vaccine and Infectious Disease Institute (VAXINFECTIO), University of Antwerp, Faculty of Medicine and Health Sciences, Antwerp, Belgium; ^2^ School of Medicine and Surgery, University of Milano-Bicocca, Monza, Italy; ^3^ Center for Cell Therapy and Regenerative Medicine, Antwerp University Hospital, Edegem, Belgium; ^4^ Virology Unit, Department of Biomedical Sciences, Institute of Tropical Medicine, Antwerp, Belgium; ^5^ Center for Oncological Research (CORE), University of Antwerp, Faculty of Medicine and Health Sciences, Antwerp, Belgium

**Keywords:** CCL4-CCR5 signaling, dendritic cell vaccination, γδ T cells, immune cell recruitment, NK cells

## Abstract

Success of dendritic cell (DC) therapy in treating malignancies is depending on the DC capacity to attract immune effector cells, considering their reciprocal crosstalk is partially regulated by cell-contact-dependent mechanisms. Although critical for therapeutic efficacy, immune cell recruitment is a largely overlooked aspect regarding optimization of DC vaccination. In this paper we have made a head-to-head comparison of interleukin (IL)-15-cultured DCs and conventional IL-4-cultured DCs with regard to their proficiency in the recruitment of (innate) immune effector cells. Here, we demonstrate that IL-4 DCs are suboptimal in attracting effector lymphocytes, while IL15 DCs provide a favorable chemokine milieu for recruiting CD8^+^ T cells, natural killer (NK) cells and gamma delta (γδ) T cells. Gene expression analysis revealed that IL-15 DCs exhibit a high expression of chemokines involved in antitumor immune effector cell attraction, while IL-4 DCs display a more immunoregulatory profile characterized by the expression of Th2 and regulatory T cell-attracting chemokines. This is confirmed by functional data indicating an enhanced recruitment of granzyme B^+^ effector lymphocytes by IL-15 DCs, as compared to IL-4 DCs, and subsequent superior killing of tumor cells by the migrated lymphocytes. Elevated CCL4 gene expression in IL-15 DCs and lowered CCR5 expression on both migrated γδ T cells and NK cells, led to validation of increased CCL4 secretion by IL15 DCs. Moreover, neutralization of CCR5 prior to migration resulted in an important inhibition of γδ T cell and NK cell recruitment by IL-15 DCs. These findings further underscore the strong immunotherapeutic potential of IL-15 DCs.

## INTRODUCTION

Active immunotherapy using tumor antigen-loaded dendritic cells (DCs) for anticancer vaccination has been under extensive investigation the past 20 years, and is currently being tested in among other phase 3 clinical trials [1]. DCs are sublime professional antigen-presenting cells, which can conveniently be manufactured from monocytes for therapeutic purposes. As a vaccine, they can enhance or induce *de novo* antitumor immune responses in cancer patients. Currently, the most widely adopted protocol generates so-called interleukin (IL)-4 DCs [2]. These monocyte-derived DCs are generated in the presence of granulocyte macrophage colony-stimulating factor and IL-4 for five days, followed by an activation step of two days with the Jonuleit cocktail consisting of the pro-inflammatory cytokines tumor necrosis factor-α, IL-1β, IL-6 and prostaglandin E2 [2]. Overall objective responses and survival benefits are, although undeniably present and comparable to some classic treatment strategies, considered rather modest [1].

This has led to the development of a new generation of DC vaccines with improved potency [3–6]. IL-15 DCs [7–10], replacing IL-4 with IL-15 for DC differentiation and using a Toll-like receptor (TLR) agonist-based maturation cocktail, have already proven themselves to outclass the conventional IL-4 DCs. This in terms of their capacity to induce both T helper (Th)1 and cytotoxic T lymphocyte (CTL) responses [7, 9–11] and to potentiate innate natural killer (NK) cell and gamma delta (γδ) T cell cytotoxicity [12, 13]. Moreover, IL-15 DCs have intrinsic cytotoxic properties, allowing them to be listed as ‘killer DCs’ [14].

To date, awareness is growing that DC-based immunotherapy should comprise more than vaccine-mediating effects on the adaptive immune system, i.e. aiming at induction of (tumor) antigen-specific CTLs and a Th1 response [15]. Tumors evading CTL recognition by downregulating major histocompatibility complex class I molecules can still be recognized and killed by NK cells [16] or γδ T cells [17]. Additionally, both cell types are important sources of pro-inflammatory cytokines, supporting *inter alia* DC and T cell functions [16, 17]. This has recently been substantiated by preclinical data showing that when innate immunotherapy is successfully applied, subsequent long-term adaptive cancer immunity is effected [18]. Therefore, IL-15 DCs could represent an improved therapeutic vaccine component with regard to immunostimulatory activity and activation of both innate and adaptive antitumor arms. However, using DCs with optimal immunocompetence is, on its own, likely not sufficient for therapeutic effectiveness. Ideally, vaccine DCs should come in contact with the necessary effector cells to perform their directing and activating functions. Whereas the capacity of DCs to migrate towards the lymph nodes is routinely assessed, less consideration is devoted to their chemoattracting properties, leaving a gap in our understanding of DC functioning. Here we investigate the attraction of immune effector cells by IL-4 DCs versus IL-15 DCs and provide new evidence of the superior immunotherapeutic potential of our short term-cultured IL-15 DCs over conventional IL-4 DCs as substantiated by DC features at the gene and protein levels, and by functional properties.

## RESULTS

### IL-15 DCs and IL-4 DCs attract distinct cell populations

**Figure 1:**
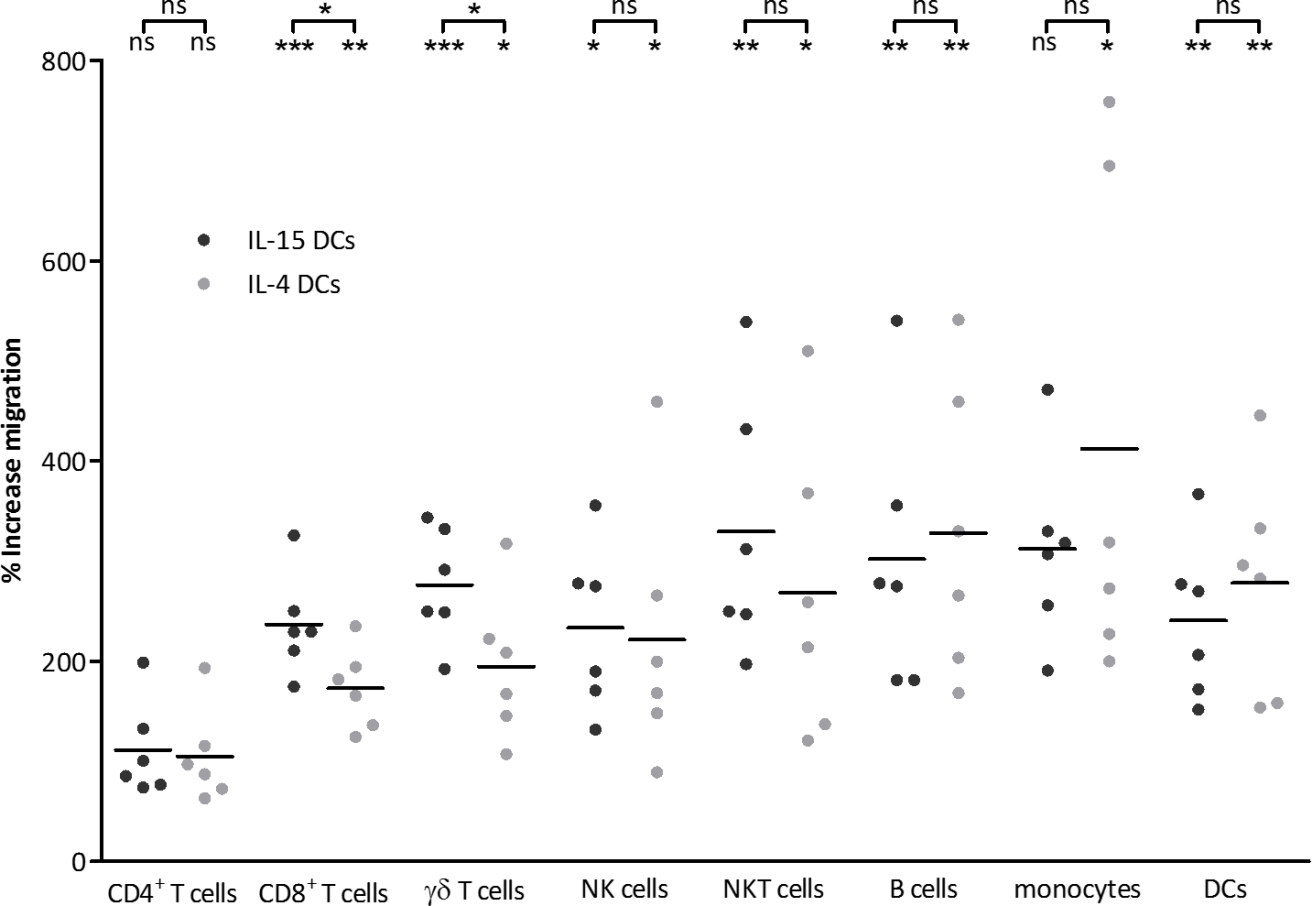
Immune effector cells are recruited by IL-15 DCs 48-hour wash-out supernatant of IL-15 DCs and IL-4 DCs was used in a three-hour 5 μm pore size transwell chemotaxis assay with PBMC. Scatter dot plots (line at mean) represent the % increase of migration, calculated as (number of migrated cells in the specific condition / number of migrated cells in the negative control) × 100, of viable migrated PBMC subsets (*n* = 6), with the negative control representing spontaneous migration towards medium. Immune cells recruited by IL-15 DC and IL-4 DC wash-out supernatant are depicted as dark grey and light grey spheres, respectively. Repeated measures ANOVA with Bonferroni's Multiple Comparison Testing was used for statistical analysis. Statistics depicted on top of the brackets represent differences between migration towards supernatant of IL-15 DCs versus IL-4 DCs, whereas statistics of migration towards each DC type as compared to the medium control is noted below the brackets. ***p < 0.001, **p < 0.01, *p < 0.05, ns, p > 0.05

To explore the migratory capacity of immune cells towards chemoattractant agents secreted by IL-15 DCs and IL-4 DCs, a three-hour migration assay towards 48-hour wash-out supernatant of autologous activated DCs was performed. Peripheral blood mononuclear cells (PBMC) were recruited by either DC preparation, corresponding with an increase of migration ± standard deviation (SD) of 190 ± 95% and 193 ± 97%, respectively (*n* = 11). Immunophenotyping of the migrated cells demonstrated that both CD8^+^ T cells and γδ T cells were significantly more recruited by IL-15 DCs in comparison with the IL-4 DCs (Figure 1 and Supplementary Figure 1A). Concerning CD8^+^ T cell migration, an increase of 236 ± 50% and 173 ± 40% was observed towards IL-15 DCs and IL-4 DCs, respectively. For the γδ T cells an increase of 276 ± 57% and 195 ± 73% was true. Both NK cells and CD3^+^ CD56^+^ γδ TCR^-^ NKT cells were attracted by IL-15 DCs and IL-4 DCs (Figure 1). This resulted in an increase of migration of 233 ± 84% and 222 ± 130% of NK cells, and 330 ± 130% and 268 ± 149% of NKT cells towards IL-15 DC and IL-4 DC wash-out supernatant, respectively. Of the evaluated cell populations, both IL-15 DCs and IL4 DCs were able to attract CD19^+^ B cells and CD14^-^ CD19^-^ CD11c^+^ blood DCs, whereas CD14^+^ monocytes only migrated towards IL-4 DC wash-out supernatant (Figure 1). CD4^+^ T cell counts were comparable in all conditions. The gating strategy of the above-mentioned subsets can be retrieved from Supplementary Figure 2.

### IL-15 DCs recruit purified γδ T cells and NK cells

**Figure 2:**
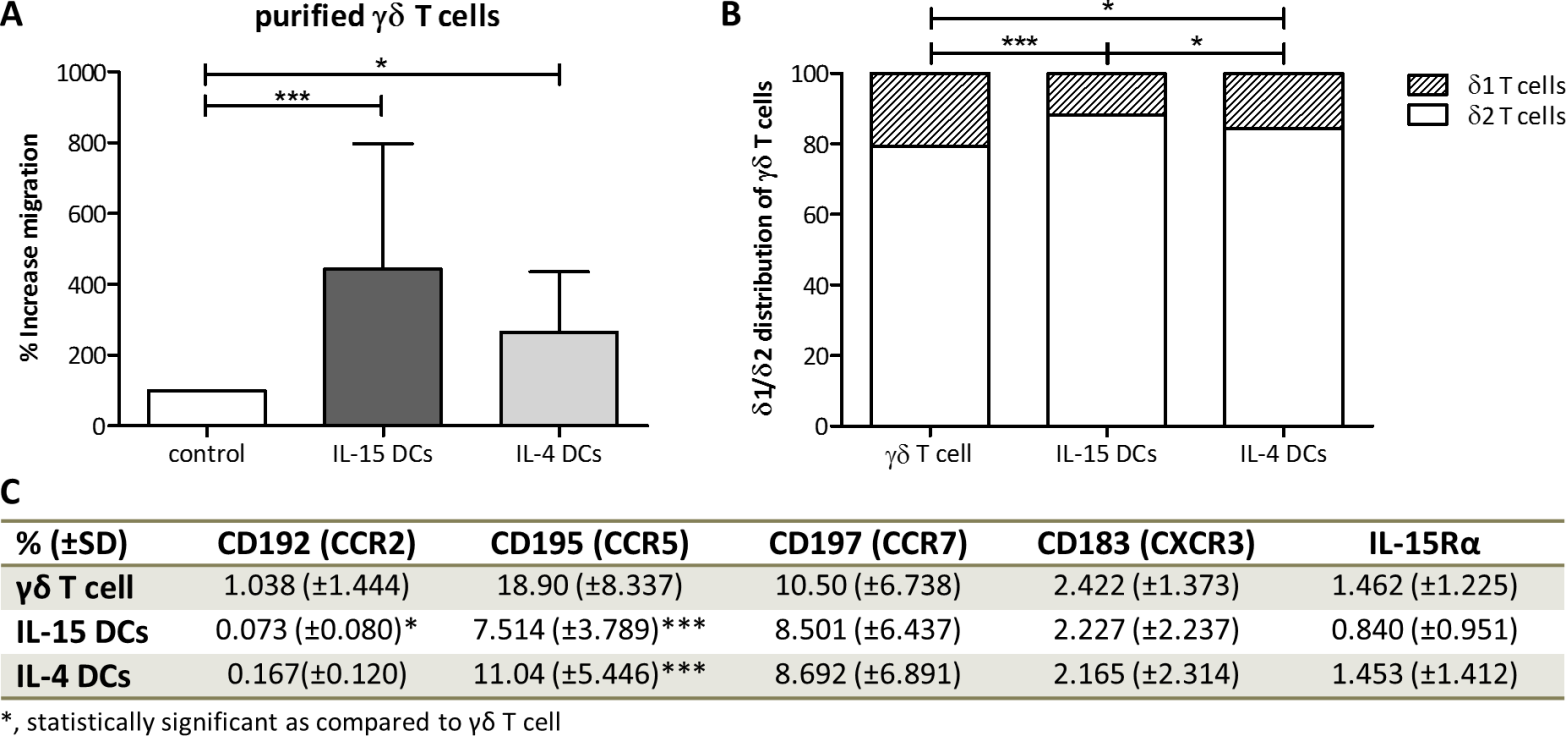
DC-mediated migration and phenotype analysis of purified γδ T cells (**A**) Percentage of isolated γδ T cell migration towards medium (control), 48-hour wash-out supernatant of IL-15 DCs (IL-15 DCs) and IL-4 DCs (IL-4 DCs). Data were calculated as percentage of migrated γδ T cells relative to background migration and show mean values (+ SD) of eleven different donors. (**B**) Bar graphs illustrate the relative distribution of Live/dead− CD3+ γδ TCR+ TCR-δ2+ and Live/dead- CD3+ γδ TCR+ TCR-δ1+ subsets of purified γδ T cells (γδ T cell) and following DC-mediated migration (n = 7). (**C**) Percentage surface expression of chemokine receptors CCR2, CCR5, CCR7 and CXCR3, and IL-15Rα on isolated Live/dead- CD3+ γδ TCR+ T cells before and after migration (n = 6–10). For δ1/δ2 distribution and CCR5/CCR7 expression) a repeated measures ANOVA with Bonferroni's Multiple Comparison Test was used, for γδ T cell migration, CCR2/CXCR3/IL-15Rα expression a Friedmann Test with Dunn's Multiple Comparison Test was used. ***p < 0.001, *p < 0.05.

**Figure 3:**
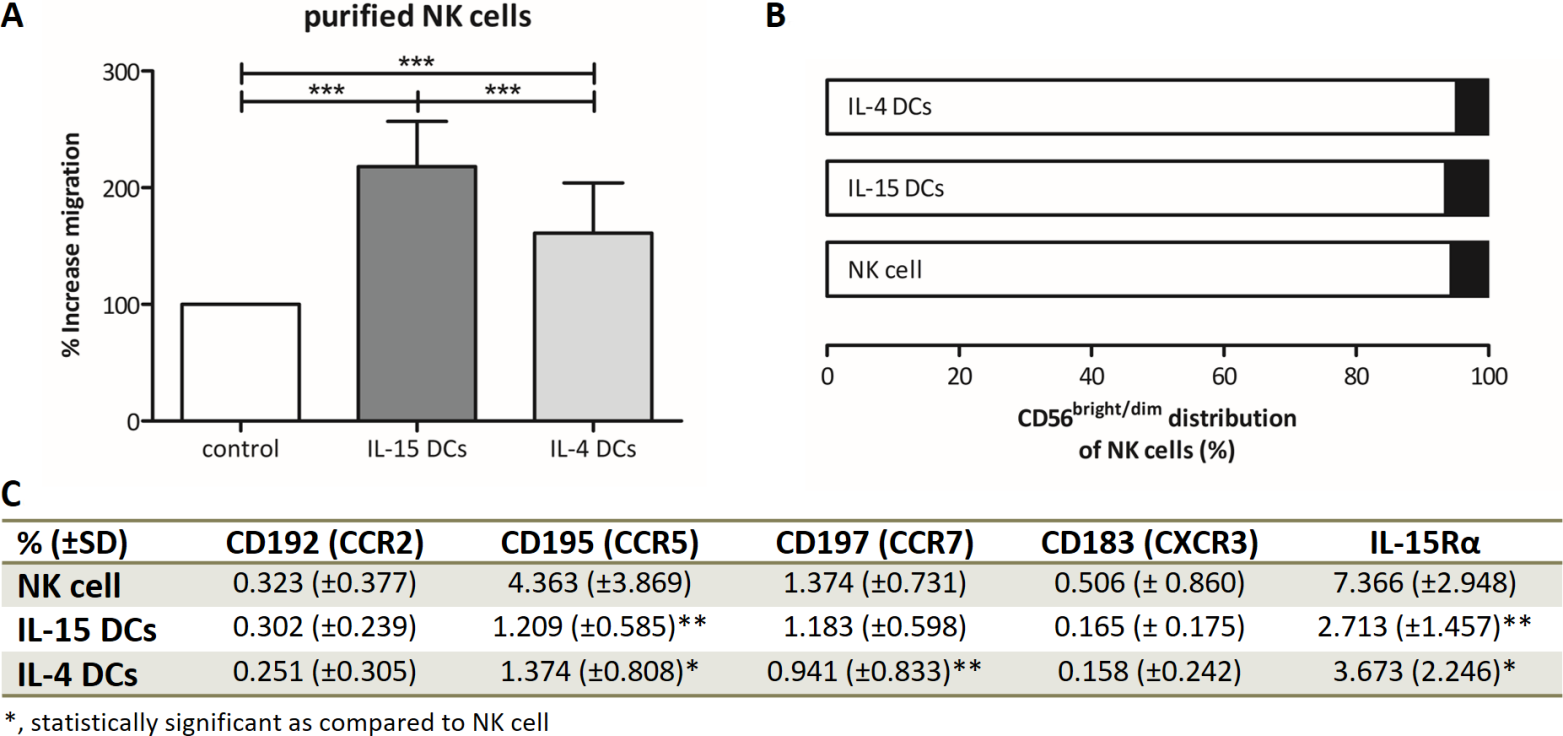
DC-mediated migration and phenotype analysis of purified NK cells (**A**) Migration capacity of purified NK cells towards the chemokine milieu of IL-15 DCs and IL-4 DCs is depicted as % increase migration (+ SD) as compared to the negative control (n = 11). (**B**) Bar graphs represent the relative distribution of Live/dead- CD3- CD56^bright^ and Live/dead- CD3− CD56^dim^ subsets in isolated NK cells prior to migration (NK cell) and in NK cells migrated towards IL-15 DC (IL-15 DCs) and IL-4 DC (IL-4 DCs) wash-out supernatant. (**C**) Percentage surface expression of chemokine receptors CCR2, CCR5, CCR7 and CXCR3, and IL-15Rα on isolated Live/dead− CD3− CD56+ NK cells before and after migration (n = 8). Repeated measures ANOVA with Bonferroni's Multiple Comparison Test (NK cell migration, IL-15Rα expression), Friedmann Test with Dunn's Multiple Comparison Test (CD56 distribution, chemokine receptor expression). ***p < 0.001, **p < 0.01, *p < 0.05.

Further elaborating on the overall higher innate effector cell recruiting potential of IL-15 DC supernatant, transwell migration assays with purified γδ T cells (Figure 2A and Supplementary Figure 1B) and NK cells (Figure 3A and Supplementary Figure 1C) support the superior chemoattractive capacity of IL-15 DCs. Unstimulated peripheral blood γδ T cells showed an increase of migration of 443 ± 354% towards IL-15 DCs, while IL-4 DCs only effectuated an increase of migration of 264 ± 172%. Both main subtypes, Vδ1 and Vδ2, of blood γδ T cells were attracted by IL15 DCs and IL-4 DCs, with a predominance of the Vδ2 fraction. This was pointedly higher after attraction by IL-15 DCs (Figure 2B). Analyzing the basal chemokine receptor profile (Figure 2C), γδ T cells were weakly positive for C-C chemokine receptor 2 (CCR2) (1.0 ± 1.4%), CXCR3 (2.4 ± 1.4%) and IL-15Rα (1.5 ± 1.2%), and positive for CCR5 (18.9 ± 8.3%) and CCR7 (10.5 ± 6.7%). After migration towards IL-15 DCs, a significantly lower percentage of CCR2 and CCR5-positive γδ T cells was observed, whereas only a lower expression of CCR5 was detected upon IL-4 DC-mediated migration. Like γδ T cells, purified peripheral blood NK cells were recruited by both IL-15 DCs (% increase of migration = 218 ± 39%) and IL-4 DCs (161 ± 43%), the latter however to a considerably lower degree as compared to IL-15 DCs (Figure 3A). Based on the CD56 surface expression, both DC types were capable of recruiting the two major NK cell subsets (Figure 3B), though a small enrichment of CD56^bright^ NK cells could be observed towards IL-15 DCs. The chemokine receptors CCR5 (6.8 ± 3.5%), CCR7 (16.6 ± 6.5) and IL-15Rα (46.0 ± 35.4) were predominantly expressed by CD56^bright^ NK cells relative to CD56^dim^ NK cells expressing 2.5 ± 0.8% CCR5, 0.9 ± 0.5% CCR7 and 5.6 ± 1.3% IL-15Rα, respectively (data not shown). Yet, expression of CCR2 and CXCR3 on purified NK cells was absent (Figure 3C). After migration a decline in surface CCR5 and IL-15Rα was observed on NK cells migrated towards IL-15 DC and IL-4 DC wash-out supernatant, and CCR7 was downregulated on NK cells recruited by IL-4 DCs (Figure 3C). Lastly, both non-migrated γδ T cells and NK cells, harvested from the transwell insert after the three-hour migration assay, displayed a comparable receptor profile relative to unstimulated peripheral blood γδ T cells and NK cells. γδ T cells and NK cells exposed to 48-hour wash-out supernatant, however, demonstrated a receptor expression profile similar to migrated γδ T cells and NK cells (Supplementary Figure 3).

### Differential chemokine gene expression

**Figure 4:**
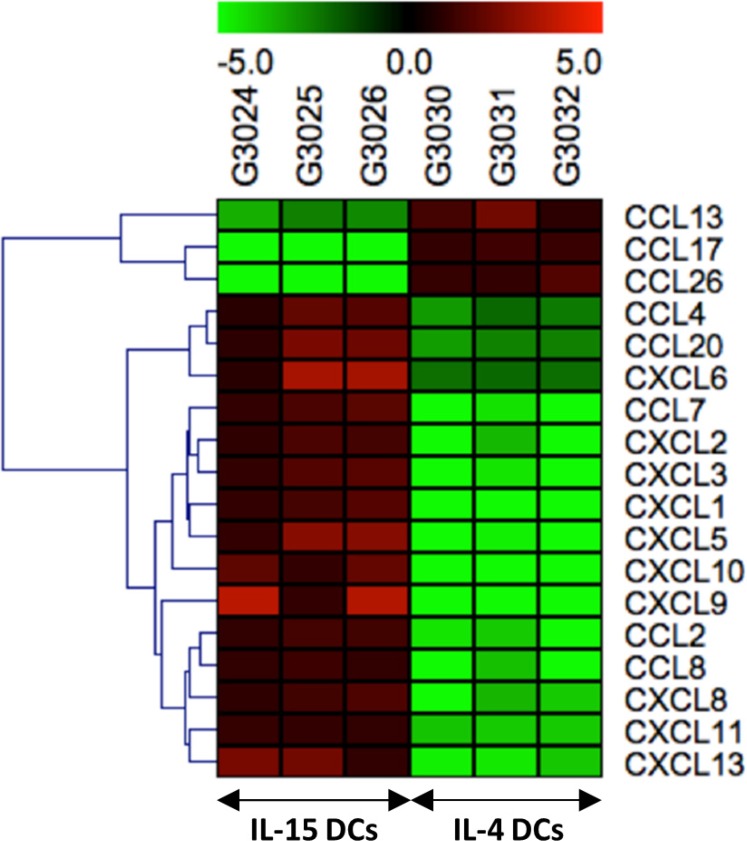
Hierarchical cluster analysis of 18 genes belonging to the chemokine family, distinctly different between IL-15 DCs and IL-4 DCs The heat map represents the differential expressed genes patterns between IL-15 DC samples (left) and IL-4 DC samples (right) of three independent donors. The value range represents the Log2 of the ratio between expression signals and the global median expression for each gene. Red color indicates up-regulated genes and green color down-regulated genes.

Gene expression analysis between IL-15 DCs and IL-4 DCs revealed a distinct dissimilitude between both activated DC types at different levels, with changes of genes involved in the chemokine signaling pathway being the most significant. Based on attraction of (innate) immune effector cells by DCs, 6 chemokine genes with differential expression between IL-15 DCs and IL-4 DCs were selected (Table 1) out of 18 genes (Figure 4) belonging to the chemokine family. Overall, IL-15 DCs exhibit a high expression of chemokines involved in antitumor immune effector cell attraction, in particular chemokine (C-C motif) ligand 2 (CCL2), CCL4, CCL7, chemokine (C-X-C motif) ligand 9 (CXCL9), CXCL10 and CXCL11, whereas IL-4 DCs display a more immunoregulatory profile characterized by high expression of Th2 and regulatory T cell attracting chemokines, notably CCL17 and CCL22.

**Table 1:** List of chemokines involved in antitumor immune effector cell attraction with differential gene expression between IL-4 DCs and IL-15 DCs

Chemokine	Chemokine receptor	Chemokine GenBank ID	Fold change	Function (references)
CCL2	CCR2CCR4	S69738	54.1	“killer DC” homing to tumor sites [67], NK cell [68], NKT cell [69] and Vδ1 T cell [70] recruitment
CCL4	CCR5	NM_002984	15.7	CD4^+^ and CD8^+^ T cell-mediated immunity [19, 37], NK cell [21] and Th1 γδ T cell [23] recruitment
CCL7	CCR2	NM_006273	117.2	recruitment activated NK cells and T cells [71, 72]
CXCL9	CXCR3	NM_002416	325.5	Th1, CD8^+^ T cell [73], NK cell [74] and NKT cell [75] recruitment
CXCL10	CXCR3	NM_001565	587.8	Th1 [41], CD8^+^ T cell [39, 73], γδ T cell [76], NK cell [44, 77] and NKT cell [78] recruitment
CXCL11	CXCR3	AF030514	31.6	Th1, CD8^+^ T cell [73], NK cell and NKT cell [42] recruitment

Fold change represents ratio in expression signal between mature IL15 DCs *vs*. IL-4 DCs. DC, dendritic cell. Th1, T helper type 1. NK cell, natural killer cell. NKT cell, natural killer T cell.

### IL-15 DC-dependent effector cell recruitment is partially CCL4-CCR5 mediated

**Figure 5:**
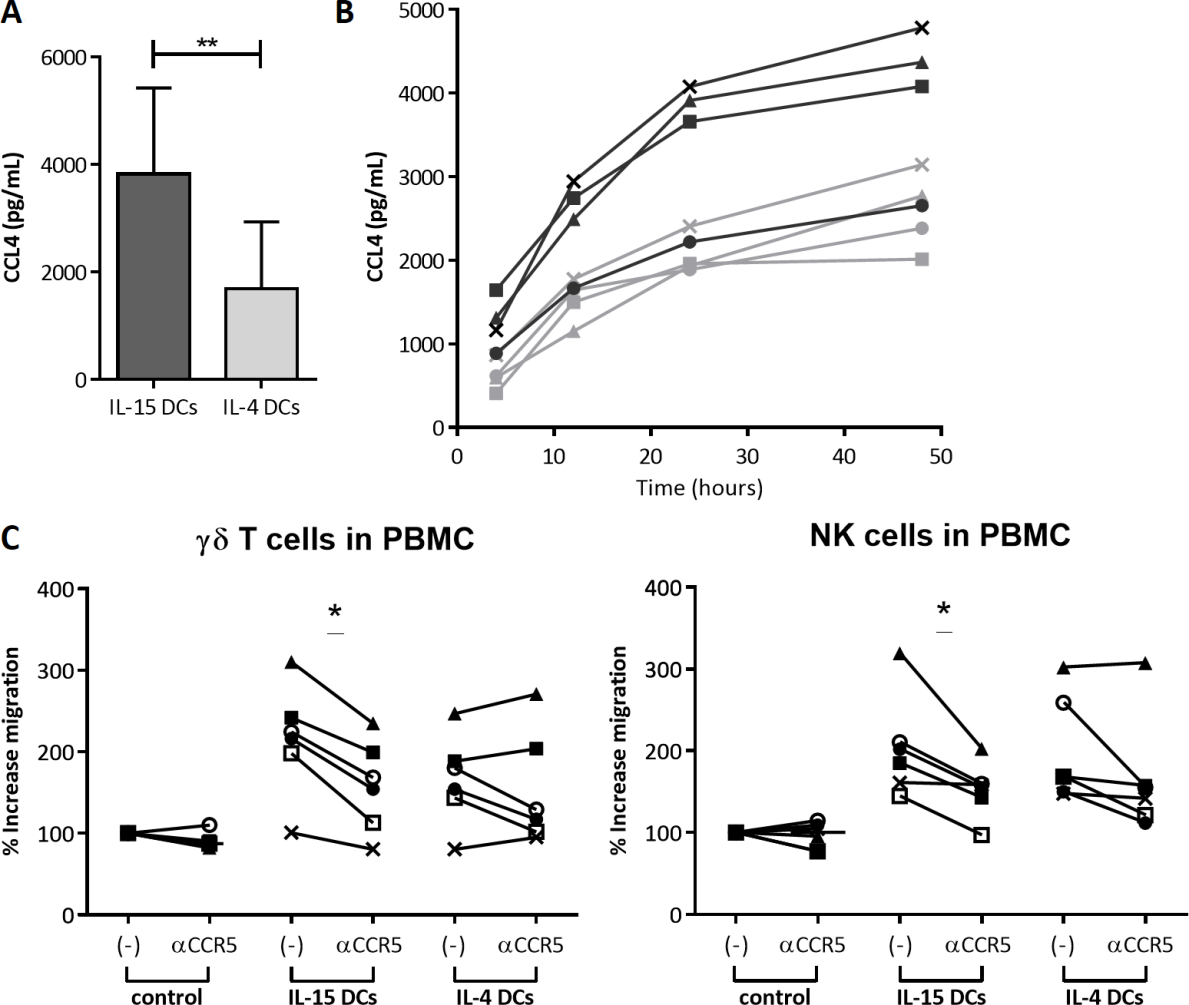
CCR5-dependent recruitment of effector immune cells by IL-15 DCs (**A**) Quantitative comparison of CCL4 secretion by IL-15 DCs and IL-4 DCs as measured by ELISA in 48-hour wash-out supernatant of IL-15 DCs and IL-4 DCs. Concentration (pg/mL + SD) is shown of duplicate conditions for 10 donors. Paired *T* test. (**B**) Time course of CCL4 secretion (pg/mL) by IL-15 DCs (dark grey lines) and IL-4 DCs (light grey lines) determined by ELISA in 4-, 12-, 24- and 48-hour wash-out supernatant (in duplicate) of 4 different donors (unique symbols). (**C**) PBMC were cultured in the absence (-) or presence of neutralizing anti-CCR5 mAbs (αCCR5) for 1 hour prior to a three-hour chemotaxis assay towards medium (control), 48-hour wash-out supernatant of IL-15 DCs (IL-15 DCs) or IL-4 DCs (IL-4 DCs). Data of 6 independent donors (unique symbols) were calculated as percentage of migrated γδ T cells and NK cells normalized to the negative control. Wilcoxon matched-pairs signed rank test. Different donors were used for experiment B and C. **p < 0.01, *p < 0.05

Taking into account the lowered CCR5 expression on both migrated γδ T cells and NK cells, the higher CCL4 chemokine gene expression in IL-15 DCs and the described effects of CCL4 on effector cells [19–23], we further evaluated the involvement of the CCL4-CCR5 axis in the chemoattractive activity of IL-15 DCs. Using a CCL4 ELISA, the higher gene expression of CCL4 in IL-15 DCs was validated at the protein level in 48-hour wash-out supernatant, demonstrating a significant higher secretion by IL-15 DCs (3819 ± 1600 pg/mL) then by IL-4 DCs (1687 ± 1244 pg/mL) (Figure 5A). CCL4 secretion kinetics indicate that the main body of CCL4 in 48-hour wash-out supernatant of both DC types accumulates in the first 24 hours after DC harvest. Still, CCL4 secretion continues after 24 hours (Figure 5B). Subsequently, we demonstrated that neutralization of CCR5 on PBMC prior to migration results in a significant inhibition of migration of γδ T cells and NK cells towards IL-15 DCs, whereas this was not observed for IL-4 DC-mediated migration (Figure 5C and Supplementary Figure 1D). It can therefore be concluded that the CCL4-CCR5 signaling pathway is to some extent responsible for the IL-15 DC-mediated effector cell recruitment.

### Cytotoxic capacity of migrated effector cells

**Figure 6:**
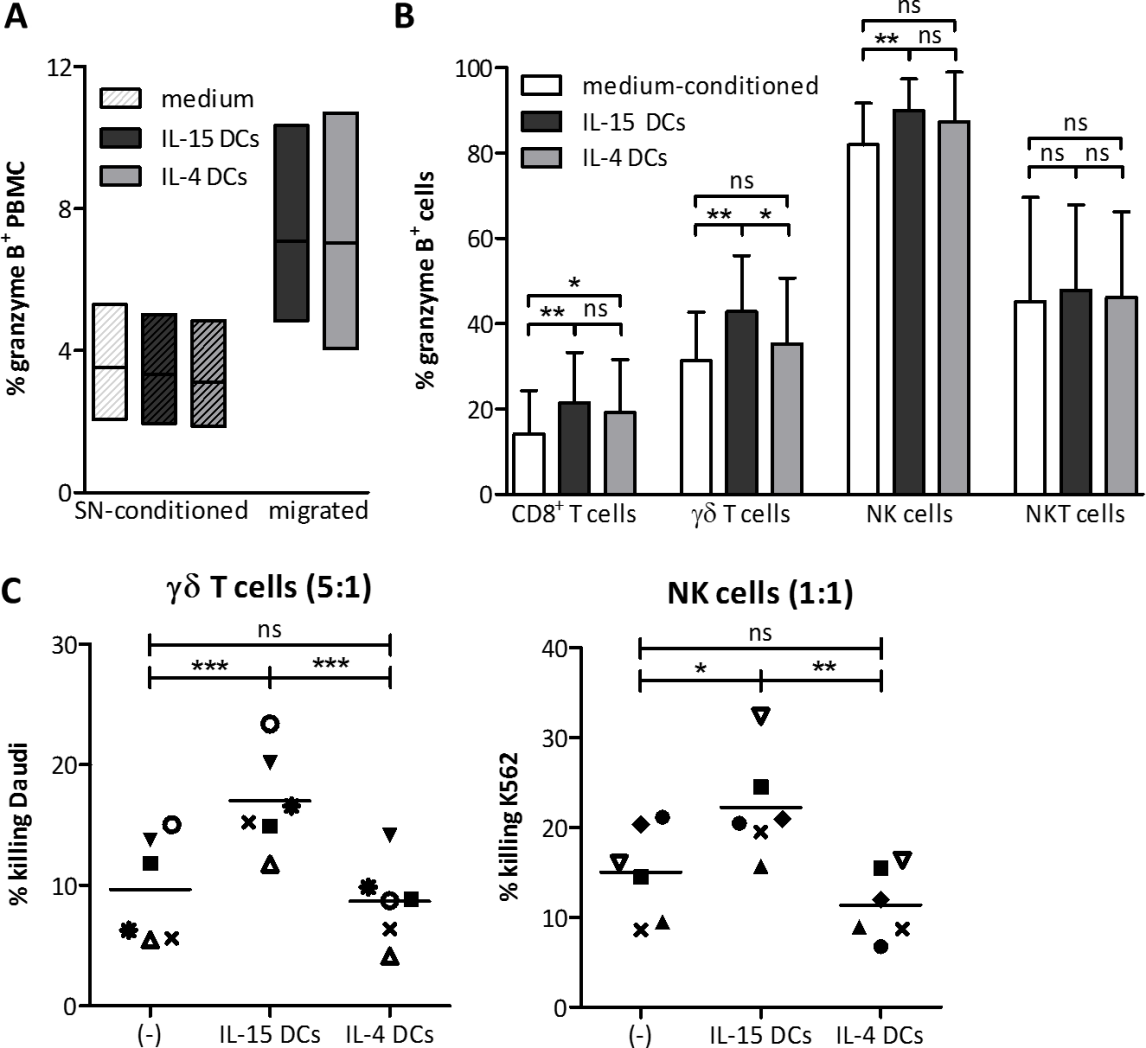
IL-15 DCs superiorly attract cytotoxic immune effector cells (**A**). Floating bars (min to max, line at mean) represent % granzyme B+ cells in PBMC (white bar), PBMC exposed for 3 hours to 48-hour wash-out supernatant of IL-15 DCs (striped dark grey bar) or IL-4 DCs (striped light grey bar), and PBMC following IL-15 DC- (dark grey bar) or IL-4 DC-mediated (light grey bar) migration (*n* = 3). (**B**) Representation of % granzyme B+ cells (+ SD) within the different effector cell subsets in full PBMC fraction (white bars) and following IL15 DC- (dark grey bars) or IL-4 DC-mediated (light grey bars) migration (*n* = 8). (**C**) Cytotoxicity was determined of migrated purified γδ T cells and NK cells towards 48-hour wash-out supernatant of IL-15 DCs (IL-15 DCs) or IL4 DCs (IL-4 DCs). Non-migrated purified cells (-) were used to define control killing capacity. Daudi and K562 target cells were added at an E:T ratio of 5:1 γδ T cells and 1:1 NK cells, respectively. Percentage tumor cell killing was determined by Annexin-V/PI staining after 4 hours and calculated using the formula specified in “Materials and methods”. Donors are represented by unique symbols (*n* = 6). Repeated measures ANOVA with Bonferroni›s Multiple Comparison Test. ***p < 0.001, **p < 0.01, *p < 0.05, ns, p > 0.5.

To evaluate the functional consequences of the different recruitment abilities of IL-4 DCs and IL-15 DCs, we first evaluated the intracellular granzyme B expression on migrated PBMC. After migration a consistent enrichment of granzyme B^+^ cells was observed towards either DC type. It concerned preferential recruitment of cytotoxic effector cells, since granzyme B expression in PBMC remained unaffected after exposure to 48-hour wash-out supernatant itself (Figure 6A). Phenotyping of the different granzyme B^+^ PMBC populations indicated a higher proportion of granzyme B^+^ CD8^+^ T cells, γδ T cells and NK cells migrating towards IL-15 DCs, whereas IL-4 DCs only significantly recruited granzyme B^+^ CD8^+^ T cells (Figure 6B). Further building on these results, we investigated whether the increased recruitment of granzyme B^+^ cells could also be translated into functional cytotoxic activity against tumor cells (Figure 6C). Performing a 4-hour cytotoxicity assay with migrated cells from a 3-hour migration assay, purified γδ T cells and NK cells attracted by secreted factors of IL-15 DCs showed a significantly higher killing capacity against tumor cells as compared to their non-migrated counterparts. Migrated γδ T cells and NK cells recruited by IL-4 DCs were equally cytotoxic as non-migrated effector cells. This supports the aforementioned (micro-array) results, stating that IL-15 DCs are superior in the recruitment of antitumor effector cells as compared to IL-4 DCs, which endorses the suitability of IL-15 DCs as a tumor vaccine.

## DISCUSSION

The past decade has witnessed the advent of a new generation of DC-based vaccines with improved potency. Herewith, IL-15 DCs represent a promising cancer vaccine, stimulating both innate and adaptive antitumor immune effector cells [11, 12, 14]. Innate immunity may be critical for activation and polarization of adaptive immune responses, and therefore for success of DC-based vaccines [16–18, 24]. Expressing CCR7 [11], IL-15 DCs have the clinical potential to migrate from the site of injection to the lymph nodes where they can stimulate antigen-specific T cells, as well as interact with NK cells and γδ T cells. However, the effectiveness of IL-15 DCs, and generally for all DC vaccines, might in the end rely on their ability to secrete the appropriate chemokines, allowing them to effectively recruit, engage, and activate (γδ) T cells and NK cells. To this extent, we made a head-to-head comparison of IL-15 DCs and conventional IL-4 DCs with regard to their proficiency in the recruitment of (innate) effector cells.

Our data show that both IL-15 DCs and IL-4 DCs are endowed with potent immune cell recruiting capacities, but attract distinct PBMC populations. First and foremost, the most common cytolytic effector cells, in particular CD8^+^ T cells, γδ T cells and NK cells, are superiorly recruited by IL-15 DCs. This is congruent with other data underpinning the weak ability of conventional IL-4 DCs to induce effector leukocyte migration [25, 26]. Both IL-15 DCs and IL-4 DCs fail to attract CD4^+^ T cells, which is, however, consistent with the *in vivo* situation in the lymph nodes. Here naïve CD4^+^ T cells approach DCs along random trajectories, without any apparent directed motion toward DCs [27]. Next, B cells are recruited by both IL-15 DCs and IL-4 DCs. The biological and clinical relevance of humoral immune responses against tumor antigens remains, however, controversial to date [28]. In light of the booked successes with monoclonal antibody-based therapeutics, to improve among other cellular antitumor immunity, attraction of B cells by DCs holds a potential benefit [29, 30]. Monocytes, on the other hand, are merely attracted by IL-4 DCs. The implications concerning DC vaccination will depend on the subtype to which these monocytes belong [31, 32], considering their dual role in cancer [33]. Interestingly, both IL-15 DCs and IL-4 DCs are able to attract autologous peripheral blood DCs. This is of importance as activated host DCs are capable of transducing the antitumor response to other immune cells, sustaining antitumor immunity [34–36].

Focusing on the cytolytic effector cells in PBMC, our microarray data show significant differences in RNA levels between IL-15 DCs and IL-4 DCs for the CD8^+^ T cell attracting chemokines CCL4 [19, 37, 38] and CXCL9-11 [39–41] suggesting their involvement in the superior attraction of CD8^+^ T cells by IL-15 DCs in comparison to IL-4 DCs. Additional functional downstream analyses are warranted to confirm their attribution. The same applies for the chemokines involved in the DC-mediated NKT cell attraction. The migratory potential of NKT cells towards DCs has so far sparsely been investigated, with only one prior report demonstrating their migratory responsiveness to the chemokine milieu of poly(I:C)-matured IL-4 DCs and to a lesser extent of conventional IL-4 DCs [42]. Since the first-mentioned DC type produces much higher levels of the CXCR3 ligands CXCL9-11, a causal relationship is possible, although no direct evidence is provided through, for example, neutralization experiments [42]. Since NKT cells are also more prone to migrate towards IL-15 DCs, as compared to IL-4 DCs, and our micro-array data suggest enhanced CXCL9-11 secretion by IL-15 DCs, it is tempting to draw a parallel between the two results. However, it is known that the majority of human NKT cells not only express the chemokine receptor CXCR3, but also CCR2, CCR5 and CXCR4 [43]. Considering we also found enhanced RNA levels of CCL2, CCL4 and CCL5 in IL-15 DCs, all ligands of the aforementioned receptors, these chemokines could therefore as well contribute to the improved attraction of NKT cells by IL15 DCs to a greater or lesser extent.

Going further into detail on the innate immune effector lymphocyte attraction by DCs, our results on DC-mediated recruitment of γδ T cells are in line with the few recently available data of Massa *et al*., demonstrating no attraction of γδ T cells by conventional IL-4 DCs [25]. IL-4 DCs stimulated with TLR3-agonist poly(I:C) or TLR4-agonist MPLA were, however, able to attract IFN-γ-producing γδ T cells. Unlike our IL-15-differentiated TLR7/8-agonist R848-matured DCs, their TLR7/8 agonist CL097-matured IL-4 DCs did not significantly recruit γδ T cells, suggesting that IL-15 during differentiation is a key requisite. Our findings on DC-mediated recruitment of NK cells are, however, inconsistent with those of Gustafsson *et al*., who could not detect migration of NK cells towards conventional IL-4 DCs [26]. Yet, when DCs were matured with poly(I:C), DC-mediated NK cell migration did occur, exemplifying involvement of TLR-engagement [26]. In line with these findings, several other research groups demonstrated NK cell migration towards TLR4-agonist (LPS)-matured DCs [44], TLR9-agonist (CpG)-stimulated plasmacytoid DCs [45] and TLR2/4-agonist *Mycobacterium bovis*-infected DCs [46]. Although one study found no significant increase in NK cell migration in response to stimulation with TLR7/8-activated plasmacytoid DC [45], a possible involvement of R848 in the IL-15 DC-induced NK cell migration cannot be precluded. R848-treated monocyte-derived DCs have previously been shown to secrete chemokines involved in NK cell migration, such as CCL2-5 and the CXCR3-binding chemokine CXCL10 [47, 48]. These chemokines can, additionally, be responsible for the increased γδ T cell recruitment. This is in line with our current findings, showing that R848-matured IL-15 DCs have an increased gene expression of CCL4 and CXCL10. Taken together, the TLR7/8 signal could prepare the IL-15 DCs for strong NK cell and γδ T cell recruitment, while IL-15 has been shown to induce secretion of γδ T cell- and NK cell-attracting chemokines by DCs (such as CCL2 [49] and CCL5 [49, 50]) and by T cells (CCL4 [51]). Differentiation with IL-15 may therefore underlie the upregulation of CCL2 and CCL4 gene expression in IL-15 DCs as compared to conventional IL-4 DCs. Furthermore, soluble IL-15, which is significantly secreted by IL-15 DCs (275.0 ± 184.5 pg/mL in 48-hour wash-out supernatant of 1 × 10^6^ IL-15 DCs ; unpublished data), has been suggested to be chemotactic for γδ T cells and NK cells [52, 53].

To further elaborate on the potential mechanisms of recruitment, the complete blood γδ T cell fraction was screened for chemokine receptor expression. We observed a high expression of CCR5 and CCR7, and a lower expression of CCR2, CXCR3 and IL-15Rα. Upon migration towards IL-15 DCs, downregulation of CCR2 and CCR5 suggests their involvement, based on ‘receptor sequestration’, a process whereby the surface expression of chemokine receptors is reduced after binding of their ligands, possible within minutes of agonist exposure [54]. Providing evidence for the presence of the respective chemokines in the wash-out supernatant and binding to their receptor, the same chemokine receptor downregulation is observed on non-migrated immune cells exposed to 48-hour wash-out supernatant as such. CCR2 expression has been associated with effector γδ T cells, whereas CCR5 have been found preferentially on Th1 cells producing interferon-γ, which is desired for an antitumor immune response [23]. CCR5 expression is restricted to Vγ9Vδ2 T cells [55], which could explain the more outspoken enrichment of this subset after migration towards IL-15 DCs. Preferential recruitment of Vγ9Vδ2 T cells would be an asset to DC vaccines in the quest of promoting DC vaccine immunogenicity for improved eradication of tumor cells [17, 56–59]. The expression of chemokine receptors on NK cells was less prominent, of which CCR5 clearest. In analogy with γδ T cells, NK cells show a significant lower CCR5 surface expression after IL-15 DC-mediated migration. While CCR5 can occur on both the CD56^bright^ and CD56^dim^ NK cell fraction, the expression is most distinct on CD56^bright^ NK cells [60, 61]. This could explain the modest enrichment of CD56^bright^ NK cells after IL-15 DC-mediated migration. Since IL-15 is capable of attracting NK cells [52], the observed downregulation of IL-15Rα could imply a contribution of this cytokine in the NK cell migration.

Here, we propose a CCL4-CCR5-dependent mechanism that underlies the superior IL-15 DC-mediated recruitment of innate and adaptive cytolytic effector cells. This interaction has been described to play a key role as a mechanism whereby immune cells organize themselves in the fight against cancer. It has been demonstrated that CCR5 is required to guide naive CD8^+^ T cells to CCL3/CCL4-secreting DC-CD4^+^ T cell complexes [38], orchestrating communication between DCs, CD4^+^ and CD8^+^ T cells in the draining lymph nodes [19]. Moreover, CCR5-dependent migration of NK cells towards TLR2/4-agonist-matured DCs has previously been described [20] and cytokine-induced killer cells use CCR5 signaling in the contact-dependent information exchange with DCs [62]. Furthermore, neutralization of CCL4 in the supernatant of virally activated plasmacytoid DCs resulted in > 80% inhibition of NK cell chemotaxis [22]. In connection herewith, we show that NK cells are recruited by IL-15 DCs, at least in part, through activation of the CCL4-CCR5 signaling pathway, and we are the first to demonstrate that γδ T cells are attracted by DCs via a CCR5-mediated mechanism.

The increased expression of CCL4 by IL-15 DCs, as compared to the IL-4 DCs, could therefore be an important and preferable element in the development of highly potent DC vaccines. Especially since the, at first glance, small differences in immune cell attraction between IL-15 DCs and IL-4 DCs have the potential to result in truly biological effects, being the recruitment of granzyme B^+^ effector lymphocytes by IL-15 DCs and subsequent significantly improved killing of tumor cells.

## MATERIALS AND METHODS

### Ethics statement and cell material

This study was approved by the Ethics Committee of the Antwerp University Hospital (Edegem, Belgium) under the reference number B300201419756. All experiments were performed using blood samples from anonymous healthy volunteers supplied by the blood bank of the Red Cross (Mechelen, Belgium). PBMC were isolated by Ficoll density gradient centrifugation (Ficoll-Paque PLUS; GE Healthcare, Diegem, Belgium) and washed in phosphate buffered saline (Life Technologies, Merelbeke, Belgium) containing 1% EDTA (Merck, Darmstadt, Germany). Positive magnetic cell selection was used for purification of CD14^+^ monocytes and γδ T cells, according to the manufacturer's instructions (Miltenyi Biotec, Amsterdam, The Netherlands), with minor modifications concerning the γδ T cell isolation protocol. Namely, the first effluent was re-administered onto the column before starting with the washing steps and these were augmented from three to five. Untouched NK cells were isolated from (CD14-depleted) PBMC using a human NK cell isolation kit (Miltenyi Biotec). Regular MACS buffer, containing EDTA, was used for all MACS separations. Untouched γδ T cells, for the functional evaluation of γδ T cells after migration, were isolated with the EasySep^™^ Human Gamma/Delta T Cell Isolation Kit (Grenoble, France), according to the manufacturer's instructions. (CD14-depleted) PBMC underwent one freeze-thaw cycle to enable the use of autologous wash-out supernatant (*vide infra*). Cryopreservation of PBMC had an insignificant negative effect on the migratory capacity (data not shown). The Burkitt's lymphoma tumor cell line Daudi was kindly provided to us by the laboratory of Prof. Kris Thielemans (Free University of Brussels, Brussels, Belgium) and the chronic myeloid leukemia tumor cell line K562 was obtained from the American Type Culture Collection (ATCC, Rockville, MD, USA; catalogue number: K-562 ATCC^®^ CCL243^™^).

### Dendritic cell culture

IL-15 DCs were prepared as per our previously reported rapid DC culture protocol [11, 12, 14]. Briefly, monocytes were seeded in Roswell Park Memorial Institute medium (RPMI; Life Technologies) supplemented with 2.5% heat-inactivated human AB serum (hAB; Invitrogen, Merelbeke, Belgium) at a final concentration of 1.0–1.2 × 10^6^ cells/mL. Differentiation was induced with 800 IU/mL granulocyte macrophage colony-stimulating factor and 200 ng/mL IL-15 (Immunotools, Friesoythe, Germany). A TLR-activating maturation cocktail, comprising R848 (3 μg/mL; Alexis Biochemicals, San Diego, USA), tumor necrosis factor-α (2.5 ng/mL), interferon-γ (250 ng/mL; Immunotools) and prostaglandin E2 (1 μg/mL; Pfizer, Puurs, Belgium), was added after 24–48 hours of differentiation for 18–20 hours. All components were bought from Invitrogen, unless stated otherwise. Control 7-day IL-4 DCs from the same blood donors were prepared as previously described in detail [11]. All subsequent experiments were performed using the obtained activated IL-15 DCs and IL-4 DCs. For the collection of x-hour wash-out supernatant, mature DCs were harvested, washed thoroughly and resuspended in fresh medium, RPMI + 2.5% hAB, at a concentration of 1 × 10^6^ cells/mL. After x hours of culturing in low absorbing polypropylene tubes, cell-free supernatant was collected and frozen at −20°C until further use.

### Cellular RNA extraction, preparation, and hybridization on microarrays

Total RNA was extracted from 7–11 × 10^6^ mature IL-15 DCs and IL-4 DCs of three independent donors using RLT lysis buffer (Qiagen, Venlo, Netherlands) according to the manufacturer's instructions. RNA quantity and purity were evaluated spectrophotometrically by Nanodrop 1000 (Thermo Scientific, Waltham, MA, USA), while the quality was assessed by the Agilent 2100 bioanalyzer (Agilent Technologies Inc, Santa Clara, CA, USA). Only samples with good RNA yield and no RNA degradation (28S:18S > 1.7 and RNA integrity number > 6) were retained for further experiments. Labelling of samples and hybridization on the Human Genome U133 plus 2.0 microarray chipswere performed according to the manufacturer's protocols (Affymetrix, Santa Clara, CA).

### Cellular microarray data analysis

The high-throughput microarray data are accessible through GEO Series accession number GSE79184. Data analysis was performed using AMDA software [63]. To filter out noise, an Inter Quartile Range > 0.2 was applied. In order to define a set of differential expressed genes, a Linear Model for Microarray Data [64] with a False Discovery Rate correction (Benjamini-Hochberg) [65] was implemented, selecting probe sets with an adjusted *p-value* ≤ 0.001 and a fold change ≥ 3 among the two conditions. Hierarchical clustering was performed through MultiExperiment Viewer [66] using the Log2 of the ratio between expression signals and the global median expression for each gene, and setting Euclidean distance as dissimilarity measure and Average linkage as linkage method.

### Cell migration assay

Transwell chemotaxis assays were performed using 24-well transwells with 5 μm pore size polycarbonate membrane. 1 × 10^6^ PBMC, 0.1 × 10^6^ purified NK cells or 0.1 × 10^6^ purified γδ T cells were seeded in the upper wells and lower wells were filled with autologous 48-hour wash-out supernatant. RPMI + 2.5% hAB in the lower compartment served as a negative control, representing the random background migration of immune cells. Cell migration was allowed for three hours at 37°C/5% CO_2_, whereupon migrated cells were collected from the lower compartment. After washing, cells were resuspended in a fixed volume and counted flow cytometrically on a FacsAria II (Becton Dickinson [BD], Erembodegem, Belgium) at a continuous flow rate. Migration was calculated as % increase of migration (number of migrated cells in the specific condition / number of migrated cells in the negative control) × 100. To examine the role of the chemokine receptor CCR5, cells were pre-incubated with neutralizing anti-CCR5 monoclonal antibodies (mAbs) (10 μg/mL, R&D; Abingdon, United Kingdom) or corresponding IgG2b isotype controls 1 hour prior to migration.

### Flow cytometric immunophenotyping

To determine circulating peripheral blood cell subtypes, migrated cells from PBMC were stained with the following mAbs: γδ TCR-FITC (Miltenyi), CD14-FITC, CD56-PE, CD3-PerCP-Cy5.5, CD11c-V450, CD8-PB (Life Technologies), CD19-APC and CD4-APC-H7. The applied gating strategy can be consulted in Supplementary Figure 2. To assess the phenotypic (chemokine receptor) profile of isolated γδ T cells and NK cells pre- and post-migration the ensuing mAbs were used: γδ TCR-FITC (Miltenyi), CD56-FITC, IL-15Rα-PE (R&D), CD195-PE-Cy7, CD3-PerCP, δ1 TCR-vioblue (Miltenyi), CD197-V450, δ2 TCR-APC (Miltenyi), CD193-APC and CD192-AF647. For evaluation of intracellular granzyme B expression, brefeldin A (Golgi-Plug 1 μL/mL; BD) and monensin (0.67 μL/mL; BD) were added to the harvested cells and incubated for 3 hours at 37°C/5% CO_2_. PBMC were then washed and incubated with surface Abs CD56-PE, CD8-PerCP, γδ TCR-APC (Miltenyi) and CD3-APC-H7 for 30 minutes at 4°C. Subsequently, cells were fixed and permeabilized, using the Foxp3/Transcription Factor Staining Buffer Set (eBioscience, Vienna, Austria), according to the manufacturer's instructions. Intracellular granzyme B-BV421 Ab was added for 1 hour at 4°C. In all applications the Live/Dead^®^ fixable dead cell stain (Invitrogen) was used to allow discrimination between viable and non-viable cells. All samples were acquired on a FACSAria II flow cytometer (BD) and mAbs were purchased from BD, unless stated otherwise. Corresponding species- and isotype-matched antibodies were used as controls.

### Cytokine secretion assay

Secretion of the chemokine CCL4 by IL-15 DCs and IL-4 DCs was determined in 1:2 diluted 4-, 12-, 24- and 48-hour wash-out supernatant of both DC types by means of a human CCL4 enzyme-linked immunosorbent assay (ELISA; Peprotech, Rocky Hill, NJ, USA), according to the manufacturer's instructions.

### Cytotoxicity assay

The killing capacity of recruited γδ T cells and NK cells was determined using a flow cytometry based protocol. Hereto, a three-hour cell migration assay (see above) with 1 × 10^6^ isolated γδ T cells or NK cells was performed, after which the cells from the lower well were harvested and counted. Target cells (Daudi or K562) were labeled with PKH67 green fluorescent cell linker (Sigma-Aldrich, Diegem, Belgium), according to manufacturer's instruction, and put together with migrated γδ T cells or NK cells at an effector:target (E:T) cell ratio of 5:1 γδ T cells or 1:1 NK cells, respectively. After 4 hour-coculture, samples were stained with Annexin V-APC (BD) and PI (BD), followed by acquisition on a FACSAria II flow cytometer (BD). Cytotoxicity was calculated based on the percentage of viable (Annexin V^−^/PI^−^) PKH67^+^ tumor cells, using the following equation: % killing = 100 % - (% viable tumor cells with effector cells/% viable tumor cells without effector cells).

### Statistics

Flow cytometry data were analyzed using FlowJo (v10; Treestar, Ashland, OR, USA). GraphPad Prism software (v5.0; San Diego, CA, USA) was used for statistical calculations, including testing to ascertain Gaussian distribution of the data, and artwork. *P*-values < 0.05 were considered statistically significant.

## CONCLUSIONS

Our results show that IL-15 DCs are superior to IL-4 DCs in terms of attraction of all important antitumor effector lymphocytes and that at least γδ T cells and NK cells exhibit enhanced cytotoxic function upon migration. Furthermore, our data demonstrate involvement of the CCL4-CCR5 signaling pathway in the improved capacity of IL-15 DCs to recruit antitumor immune effector lymphocytes, by means of increased expression and secretion of CCL4 by IL-15 DCs. In addition to the previously demonstrated superior T cell- and NK cell stimulatory properties and direct tumor cell killing capacity of IL-15 DCs [11, 12, 14], these findings further underscore their strong immunotherapeutic potential as next-generation DC-based vaccines.

## SUPPLEMENTARY MATERIALS FIGURES


