# Supplementary material for: Desirable cytolytic immune effector cell recruitment by interleukin-15 dendritic cells
Source: Oncotarget. 2017 Jan 13;8(8):13652–65. doi: 10.18632/oncotarget.14622 (PMC5355127; doi:10.18632/oncotarget.14622)
Supplement: Supplementary file 1 [file oncotarget-08-13652-s001.pdf]

Desirable cytolytic immune effector cell recruitment by interleukin-15 dendritic cells

Supplementary Materials

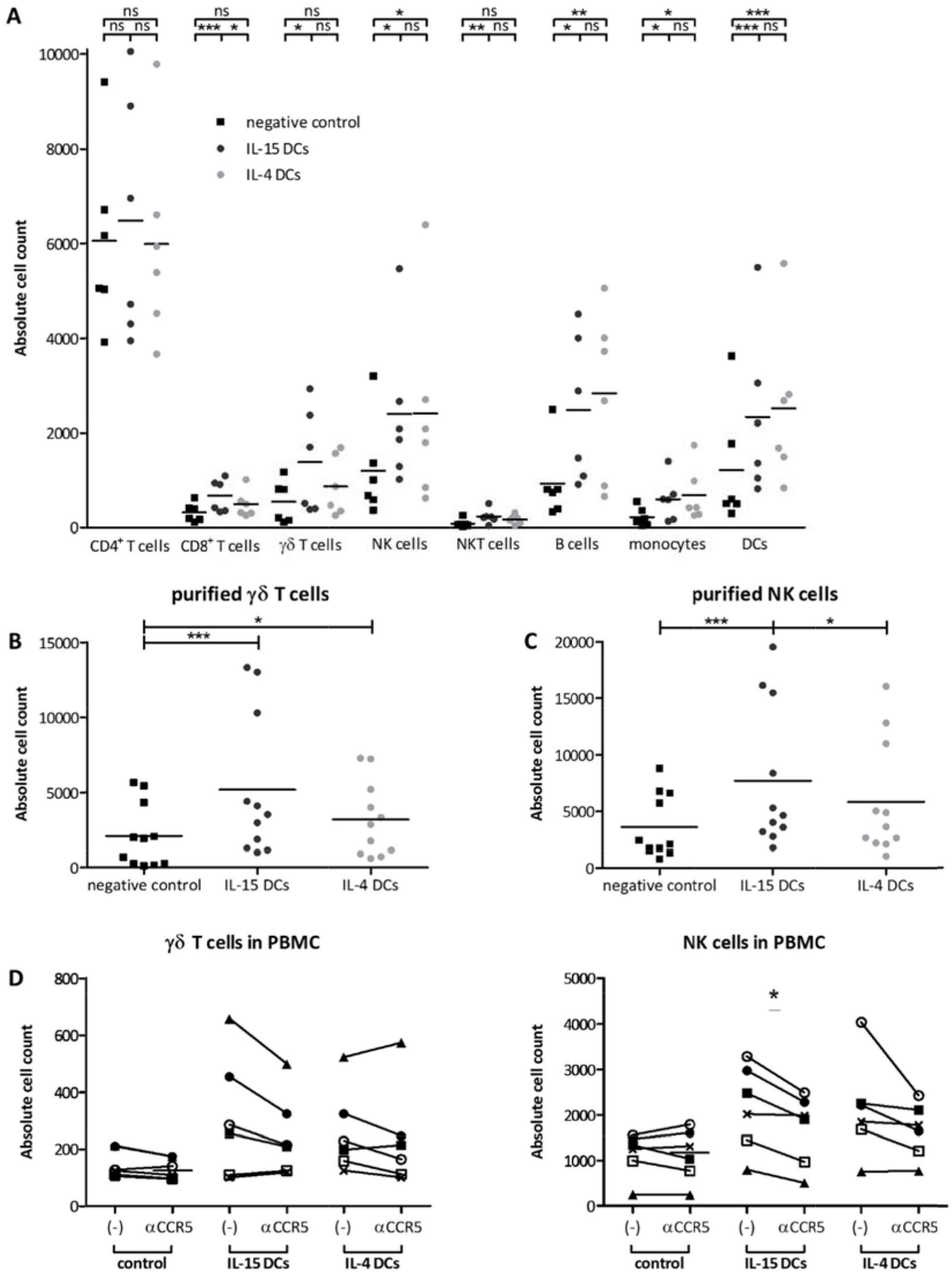

Supplementary Figure 1: Graphic display of absolute cell counts as an adjunct to the figures depicting % increase of migration. Absolute cell count representation of (**A**) Figure 1, (**B**) Figure 2A, (**C**) Figure 3A and (**D**) Figure 5C.

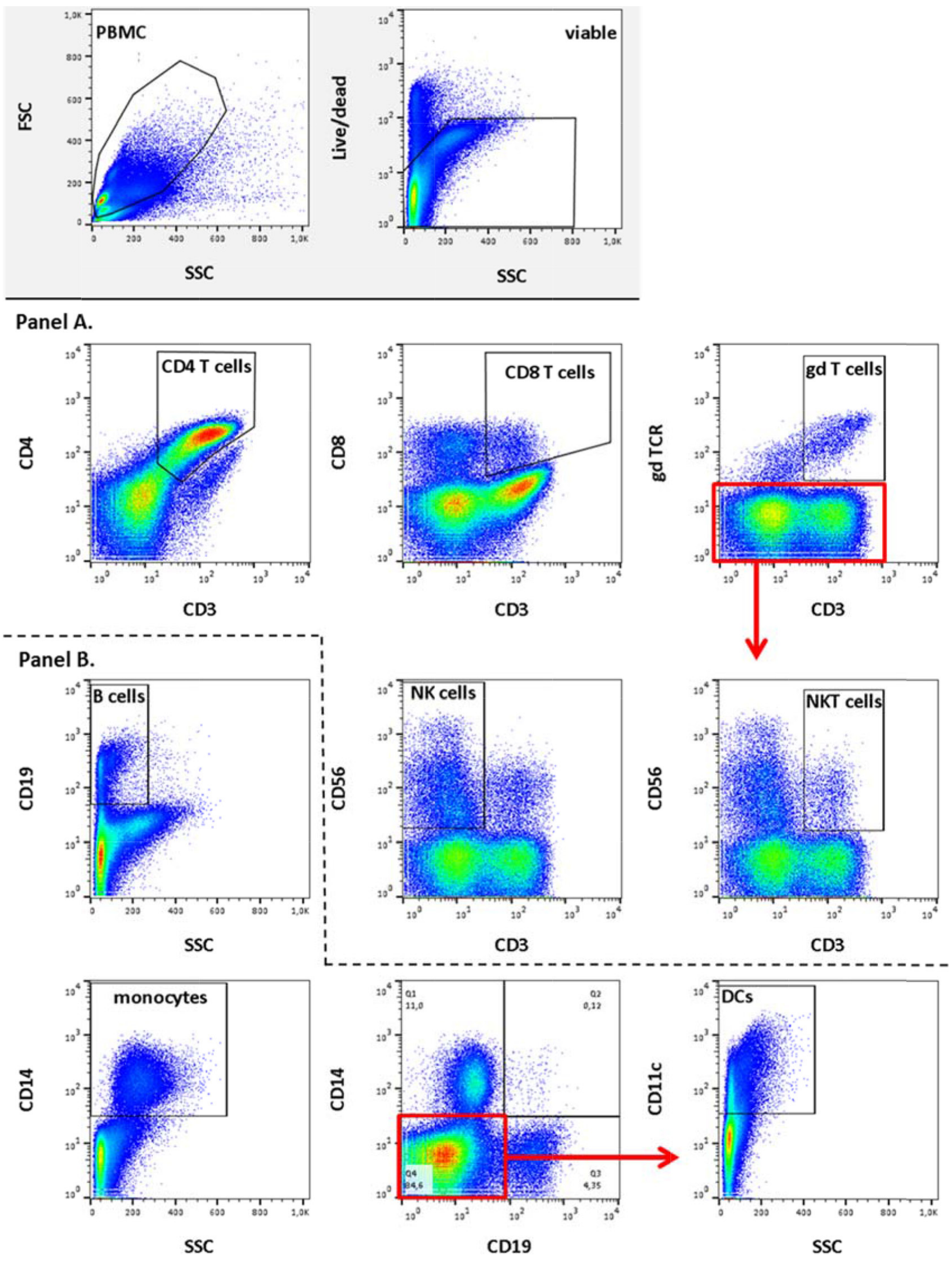

Supplementary Figure 2: Gating strategy for determination of cell subsets within unfractioned PBMC. Initial gating is done on FSC and SSC, eliminating debris, followed by the exclusion of dead cells. Panel A) Within the viable cell gate, $CD4^+$ T cells ($CD3^+CD4^+$), $CD8^+$ T cells ($CD3^+CD8^+$), γδ T cells ($CD3^+$ γδ TCR^+), and NK cells ($CD3^-CD56^+$) are identified. Subsequently, NKT cells are defined as $CD3^+CD56^+$ in the γδ TCR^- gate. Panel B) Viable monocytes are $CD14^+$, B cells are $CD19^+$ and dendritic cells (DCs) are selected as $CD11c^+$ cells within the $CD14^-CD19^-$ gate.

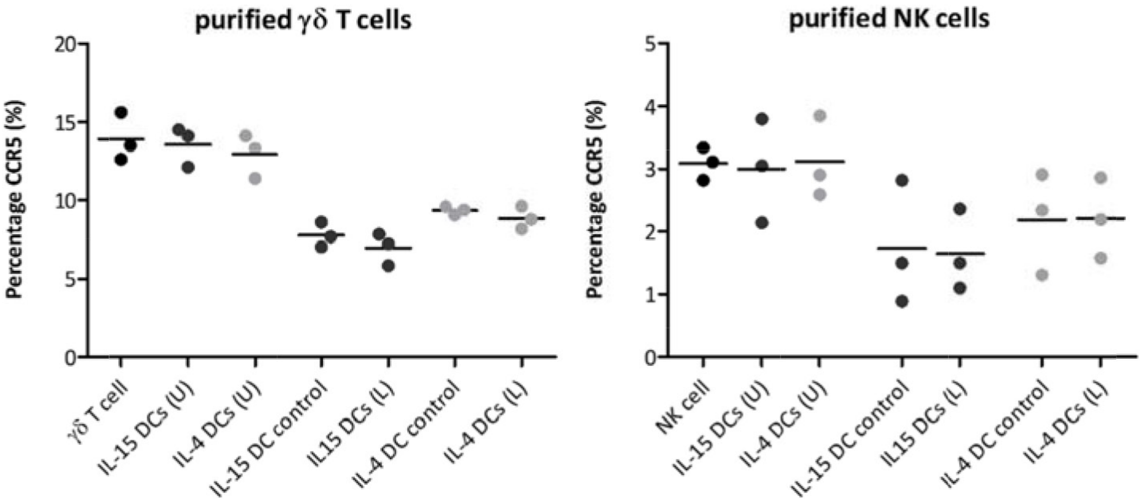

Supplementary Figure 3: Influence of DC supernatant on purified γδ T cell and NK cell CCR5 expression. Percentage surface expression of CCR5 was assessed flow cytometrically on isolated viable $CD3^+$ γδ TCR^+ T cells (γδ T cell) and $CD3^-CD56^+$ NK cells (NK cell), immune cells exposed for 3 hours to 48-hour wash-out supernatant of IL-15 DCs (IL-15 DC control) or IL-4 DCs (IL-4 DC control), and immune cells harvested from the lower well (L) or transwell insert (U) after a three-hour migration assay towards IL-15 DC (IL-15 DCs) or IL-4 DC (IL-4 DCs) wash-out supernatant.
